# Evaluating institutional capacity for research ethics in Africa: a case study from Botswana

**DOI:** 10.1186/1472-6939-14-31

**Published:** 2013-07-30

**Authors:** Adnan A Hyder, Waleed Zafar, Joseph Ali, Robert Ssekubugu, Paul Ndebele, Nancy Kass

**Affiliations:** 1 Johns Hopkins Bloomberg School of Public Health, 615 North Wolfe Street, Suite E-8132, Baltimore, MD 21205, USA; 2 Johns Hopkins Berman Institute of Bioethics, Baltimore, MD, USA; 3 Rakai Health Sciences Program, Uganda Virus Research Institute, Kalisizo, Uganda; 4 University of Botswana, Francistown, Botswana

**Keywords:** Africa, Botswana, Research ethics, Bioethics, Capacity development

## Abstract

**Background:**

The increase in the volume of research conducted in Low and Middle Income Countries (LMIC), has brought a renewed international focus on processes for ethical conduct of research. Several programs have been initiated to strengthen the capacity for research ethics in LMIC. However, most such programs focus on individual training or development of ethics review committees. The objective of this paper is to present an approach to institutional capacity assessment in research ethics and application of this approach in the form of a case study from an institution in Africa.

**Methods:**

We adapted the Octagon model originally used by the Swedish International Development Cooperation Agency to assess an organization along eight domains in research ethics: basic values and identity; structure and organization; ability to carry out activities; relevance of activities to stated goals; capacity of staff and management; administrative, financing and accounting systems; its relations with target groups; and the national context. We used a mixed methods approach to collect empirical data at the University of Botswana from March to December 2010.

**Results:**

The overall shape of the external evaluation Octagon suggests that strengths of the University of Botswana are in the areas of structure, relevance, production and identity; while the university still needs more work in the areas of systems of finance, target groups, and environment. The Octagons also show the similarities and discrepancies between the 'external' and 'internal' evaluations and provide an opportunity for exploration of these different assessments. For example, the discrepant score for 'identity' between internal and external evaluations allows for an exploration of what constitutes a strong identity for research ethics at the University of Botswana and how it can be strengthened.

**Conclusions:**

There is a general lack of frameworks for evaluating research ethics capacity in LMICs. We presented an approach that stresses evaluation from both internal and external perspectives. This case study highlights the university's rapid progress in developing research ethics capacity and points to some notable areas for improvement. We believe that such an empirically-driven and participatory assessment allows a more holistic measurement and promotion of institutional capacity strengthening for research ethics in LMICs.

## Background

The volume of research in Low and Middle Income Countries (LMIC) has increased significantly during the last two decades [1]. According to one estimate, the number of investigators regulated by the U.S. Food and Drug Administration who are based outside the US has grown at an annual rate of 15% since 2002 [2]. Almost one third of industry sponsored phase three clinical trials are conducted solely outside the US, of which a large number are conducted in LMIC. A review of clinical trials reported in three of the top medical journals (New England Journal of Medicine, Lancet, Journal of American Medical Association) found that between 1995 and 2005, the number of countries serving as trial sites outside the US more than doubled with the greatest increase seen in Africa, the Middle East and Eastern Europe [1]. Similar trends are reported in social, behavioral and health policy research in LMIC [3,4].

Concerns, however, have been raised regarding the ethics of research in LMIC [5]. One set of concerns about "globalization of clinical research" center on autonomy and the potential for exploitation of research participants. Adequacy of informed consent, possibility of undue influence through higher payments in multinational trials, and the discrepancy in standards of care between high-income countries and LMIC are other issues that have been raised [6]. Another set of concerns focus on transparency in the conduct of trials and the ability of institutional review boards (IRBs) or research ethics committees from wealthier countries to oversee trials primarily conducted in LMIC [7]. Finally, there are concerns regarding equitable access of LMIC populations to the fruits of health research, in addition to the expectation from researchers to provide "ancillary care" for medical problems unrelated to the research study [8,9].

Research ethics systems in LMIC need resources, personnel trained in research ethics, and an ability to apply principles of ethical review to local needs and settings. Several programs have been initiated to strengthen the capacity for research ethics in LMIC [10]. One example is the International Bioethics Education and Career Development Award launched in 2000 by the Fogarty International Center of the National Institutes of Health. The objectives of this program are to improve the quality of international ethics training, to support advanced long-term training of professionals in developing countries, and to develop intensive short courses for individuals involved in the ethical review of human participant research [11]. The Johns Hopkins Fogarty African Bioethics Training Program (FABTP) was initiated through this funding mechanism and, for the last 11 years, has conducted training for professionals from sub-Saharan Africa in research ethics [12].

Other strategies to increase capacity in the area of research ethics also have emerged in the last decade. The World Health Organization has launched the Strategic Initiative for Developing Capacity in Ethical Review aimed at developing culturally sensitive capacity for ethical review of human research in various parts of the world [13]. The Wellcome Trust also initiated several fellowships aimed at training health care professionals in LMIC in biomedical research ethics [14]. The European and Developing Countries Clinical Trials Partnership has in the past few years launched grants aimed at the establishment or strengthening of IRBs in African countries [15].

While such programs are important in developing the capacity for research ethics in LMIC, they have generally focused on individual training or training of IRBs. Less attention, however, has focused on the critical area of developing institutional capacity more broadly in research ethics and in strengthening research ethics systems as a whole [10]. The Association for the Accreditation of Human Research Protection Programs of the United States has described the research ethics system in terms of five domains: legal authorities, institutions, researchers, ethics review committees, and participants. According to this, it is important to examine whether policies and procedures of the organization as a whole create a coherent, effective scheme for the protection of research participants [16]. Hyder et al. suggested that institutional commitment to research ethics also goes beyond the training of individuals or IRBs and is characterized by: protection of participants from research related risks; promotion of ethical conduct within the institution; establishment of appropriate institutional priorities, organizational structures and procedures; and conformity with national and regional laws and guidelines [10].

A first step toward development of institutional capacity is the conduct of an organizational needs assessment for research ethics. Institutional capacities in LMICs are highly variable and call for a flexible approach regarding methods of data collection and their analysis. The overall goal of this paper is to present an approach we developed for evaluating institutional research ethics systems in LMIC. We first briefly describe our training program and then introduce our proposed approach to institutional evaluation for research ethics capacity that uses eight domains. We present a case study from Botswana applying this assessment, and then discuss the substantive outcomes, as well as the need to promote more global dialogue in institutional strengthening for research ethics. We hope that our approach can be tested in other contexts, and also help inform research ethics capacity development efforts.

### Fogarty African Bioethics Training Program (FABTP)

Since its inception in 2001, the Johns Hopkins-Fogarty African Bioethics Training Program (FABTP) has trained 30 professionals, scientists, and senior scholars from 14 countries across sub-Saharan Africa. Prior to 2010, the program provided a one-year opportunity for qualified individuals to participate in intensive bioethics and research ethics training at Johns Hopkins Berman Institute of Bioethics and Bloomberg School of Public Health, with additional workshops, seminars, and IRB activities at the National Institutes of Health and Georgetown University [12]. Following the six month training period, trainees returned to their home countries to conduct independent projects related to research ethics.

In 2010, FABTP shifted its focus to initiate annual collaborative partnerships with African institutions in an effort to enhance research ethics capacity within selected institutions. In addition to one year of bioethics and research ethics training at Johns Hopkins University (JHU) for two individuals from the selected institution, FABTP also provides one-month tailored training opportunities in research ethics for two additional partner faculty, investigators, ethics committee members, or staff. FABTP also "sponsors a training" workshop in research ethics each year, hosted at the African institution, and co-taught by JHU and partner faculty as well as program alumni. Further, under the institutional partnership model, FABTP engages in on-site and virtual consultations with institutional leadership to assist with evaluating and enhancing the integration of research ethics into organizational structures. This also includes technical assistance with the development of research ethics centers, units or programs. Program participants are offered mentorship from JHU faculty and are provided with networking opportunities for their continued professional and institutional development.

## Methods

### An approach to institutional assessment

Several models for the assessment of institutional *research* capacity have been described [17-21]. However, most of these models have been developed from a high-income country perspective and cannot be easily adapted for evaluation of *research ethics* capacity in LMIC. A system for evaluating research ethics capacity in LMIC presumably would allow multiple elements of a research ethics system to be assessed; would be pragmatic in terms of timeliness for a LMIC context; and allow for empirical testing. In pursuing such an approach, we adapted the *Octagon model* developed by the Swedish International Development Cooperation Agency that was intended as a rapid assessment instrument for the strengths and weaknesses of non-governmental organizations [22]. The octagon assesses an organization along eight domains: basic values and identity; structure and organization; ability to carry out activities; relevance of activities to the stated goals; capacity of staff and management; administrative, financing and accounting systems; relations with target groups; and relationship of the organization with the larger environment in which it is operating. The octagon offers a simple yet deliberative, and iterative tool for institutional assessment over time.

In our adaptation of the Octagon model to the context of research ethics in LMIC, the category of *basic values and identity* is applied to determine whether the institution has clearly articulated aims and objectives for research ethics and whether it has formulated strategies to achieve those objectives. Highest points are awarded if these aims and objectives are available in a written form and if there are clearly articulated strategies to achieve these objectives. The *Structure and management* domain of the assessment deals with how the organization is structured and how its activities are organized for research ethics. Specifically it looks at whether the research ethics duties and responsibilities are clearly defined for everyone working for the organization and whether it abides by the principles of transparency, accountability and democratic rules. Highest points in this category are awarded if there is an organizational chart showing how different responsibilities are divided and the decision making for research ethics within the organization is transparent.

Under the domain of *Implementation*, the organization is evaluated on its ability to systematically carry out its research ethics activities through an operational plan. In addition, the organization is evaluated on whether it seeks feedback on its current operations and whether it learns from its experience. The organization’s activities are evaluated for their *correspondence with the organization’s stated goals* for research ethics under the fourth domain. Under this category two related issues are evaluated: whether the staff members have the requisite *skills* to perform their research ethics duties, and whether the management enjoys the confidence of the workers.

The category of *financing and administration* involves evaluation of the organization's sources of financing research ethics activities and the processes used to account for these funds. High points are awarded to organizations that are able to obtain funds from several sources including through fees and research grants. In addition high points are awarded to organizations that have transparent and professional accounting practices. Every organization may have research ethics activities targeted towards a particular group of people; therefore the seventh category involves evaluation of whether the organization has a clear conception of the types and nature of *target groups*. In this category, the organization's perceived legitimacy in research ethics among those target stakeholders is also assessed. Finally, the organization’s activities are assessed within the external ethics *regulatory context* provided by the government and the national health research system; and high points are given for leadership at national level and consistency with national policies. Table 1 provides a summary of all eight domains.

**Table 1:** Description of the octagon framework as applied to the research ethics system at an institution

	**Domain**	**Components**	**Description**	**Highest points are awarded if**
1	Basic values and identity	•Formulation of organization's vision and mission	Documents that describe reasons for the establishment of the organization, objectives the organization wishes to achieve in research ethics in the future (vision), and contribution the organization wishes to make in research ethics (mission).	•Organization's vision and mission are documented in writing, are known and accepted by all members of staff.
		•Formulation of relevant strategies		•The organization has devised strategies that have been documented and which are clearly linked to the organization's vision
2	Structure and organization of activities	•Application of a clear division of duties and responsibilities for research ethics	Duties and responsibilities are allocated and coordinated; democratic rules are applied and these rules manifest in the organization's constitution or strategic plan or rules and regulations; decision-makers can be held responsible for their decisions and actions.	•Management and staff know the duties, responsibilities and powers they have for research ethics
		•Application of democratic rules		•Transparent routines and systems for approval of accounts and reports, and for scrutiny of decisions made
				•These systems include participation of both men and women.
3	Implementation of activities / Production	•Planning for the implementation of activities for research ethics	What are the research ethics outputs the organization has identified and can the organization describe its research ethics activities in the form of operational plans? Are the plans useful for the implementation of research ethics activities?	•Operational plans that are actually used by management and results achieved have been documented.
		•Follow-up and learning from work done		•Systems for regular follow-up and for making good use of experience gained.
4	Relevance	•Content of research ethics activities corresponds with the vision/mission	Whether the organization’s research ethics activities in their content and methods correspond to its goal and vision/mission.	•Activities of the organization actually correspond to its vision/mission
		•Working methods correspond with the vision/mission		
5	Right skills in relation to activities / Competence	•Professional qualifications and experience of the staff	Whether the organization has a recruitment strategy and selects personnel in accordance with existing, documented criteria for research ethics.	•Job descriptions for all posts, and staff in place that fully meet the criteria of the job descriptions.
		•Ability of management		•The staff regards management as legitimate and gives management its active support.
6	Systems for financing and administration	•Administration of financial resources	Sources of finance for research ethics, whether financial resources are sufficient for planned activities and whether there are plans to reduce dependence on external grants. Examine routines for systematic documentation of activities	•Guaranteed financing and several sources of funds for research ethics.
		•Administrative routines		•Efficient administrative systems in which documents are filed systematically

7	Target groups	•Support and acceptance by target groups; dialogue with target groups on research ethics	Whether the organization encourages the continuous and broad participation of the target groups in its research ethics activities.	•Organization has documented how the target groups are defined.
				•Target groups are clearly involved in activities.
		•Legitimacy for its work, and active participation in networks		
8	Working environment	•Government rules / regulatory environment for research ethics	What is the legal context in which the organization operates, and what rules or framework does the government have regarding research ethics.	•Activities are in line with the government's vision and policy statements of multilateral organizations e.g. the World Health Organization
		•Influence on national health research system		
				•Plays a leadership role at national level
				

Source: Octagon model modified from Swedish International Development Cooperation Agency.

Each of these eight domains is rated on a 7-point scale, where 1 is lowest and 7 is the highest score (Table 2). Scores on all eight domains are then mapped along each of the eight angles of an "octagon". Evaluation scores are developed by each rater independently and then a consensus score was developed after discussion among the teams, separately for both external and internal evaluations (Table 2). The octagon has several advantages including creation of a visual summary (on MS Excel), the ability to track progress over time since these octagons can be superimposed over each other to create a graphic of how institutional capacity changes over time, and comparison of different domains within the organization. However, the evaluation is dependent on the time it is conducted, the team conducting it and is primarily meant for tracking progress rather than an absolute assessment. The Octagon model can be informed by using different methods to study institutions; we developed an operational approach that is described below and was pilot tested in Botswana.

**Table 2:** Sample from scoring guide for raters in the internal or external teams

**Excellent**	**Very good**	**Good**	**Reasonable**	**Weak**	**Very weak**	**Non-existent**
**7**	**6**	**5**	**4**	**3**	**2**	**1**
**Main aspects**		**Detailed aspects**		**Description**		**Score**
Relevance		1. The content of activities correspond with the vision		Ascertain highest points are awarded if.		
		2. Working methods correspond with the vision		*The activities of the organization actually correspond to its vision and this is the subject of continuous reflection and internal discussion.		
				*The organization practices what it preaches.		
				Lowest points are awarded if.		
				*There is no link between the origination’s activities and its vision, and planning and methods development are not given priority.		
				*There are double standards and self-contradiction in the organization.		
Right skills in relation to activities		1. Professional qualifications and experience of the staff		Ascertain whether the organization has a recruitment strategy and selects personnel in accordance with existing, documented criteria.		
		2. Ability of management		Highest points are awarded to organizations.		
				*That have documented job descriptions for all posts and which, in addition, have staff in place that fully meet the criteria of the job descriptions.		
				*If the staff regard management as legitimate and give management their active support.		
				Lowest points are awarded to organizations.		
				*In which there are no documented requirements of qualifications and experience.		
				*Where management is not legitimate in the eyes of the staff or does not participate in activities.		
Working environment		1. Government rules / regulatory environment		Ascertain what the legal context is in which the organization operates, and what rules or frameworks the government has regarding the organization's area of activities.		
				Highest points are awarded if the organization's.		
				*Activities are in line with the government's vision and policy statements of multilateral organizations e.g. WHO.		
				*Plays a leadership role.		
				Lowest points are awarded if the organization's activities fall short of the government policies.		

Please rate the 8 main aspects of your organization’s status of research ethics on the following scale.

Our approach was to operationalize the Octagon model by developing a *mixed methods* approach to collecting data and populating the eight domains. A single questionnaire to capture, broadly, an institutional assessment of research ethics capacity was developed to be completed by institutional leaders (e.g. Dean, Principal, Vice Chancellor). Institutional leadership was instructed that several individuals might be involved in completing different sections of this questionnaire based on their specific expertise. This institutional survey was based on previous work done by the World Health Organization (WHO) on assessment of national health research systems, and similar conceptual and empirical work conducted by the authors [23,24]. The structured survey comprised four modules (related to institution policies, research departments, institutional review boards and national policies) with 194 questions (Table 3). The modules cover key conceptual domains pertinent to research ethics systems and questions on the survey significantly map onto the 8 domains of the Octagon model.

**Table 3:** Sample questions from the institutional survey for research ethics system

**Domains**	**Components**	**Illustrative questions**
Institution	Institutional leadership, plans and policies	Does institution has written policies that clearly state the kinds of research protocols that must be submitted to the IRB?
	Institutional finances	Does the institution set aside financial support for the Office of Research?
	Formal teaching of research ethics	Does institution offer any type of educational opportunities in research ethics for your students?
	Human resources	Are there individuals currently on institution's faculty/staff with a significant academic interest in bioethics?
	Student dissertations or theses policies	Is there an institution-wide requirement that student proposals involving research with human beings discuss any potential ethics issues?
	External collaborations	Does institution have any significant ongoing external collaboration in research?
Office of Research and Development (or equivalent office)	Structure	Does institution have an organizational chart showing where the Office of Research sits in relation to other departments?
	Finances	What approximate percentage of the institution's budget is devoted to research ethics activities?
	Personnel and resources	Please indicate staff positions and primary activities of the office?
	Training activities	Is any professional training required for research ethics personnel?
Institutional Review Boards – IRB (or Research Ethics Committees)	History and function	When was the present IRB inaugurated?
	Registration	Is the IRB registered with the US Office for Human Research Protection?
	External collaborations	Does IRB itself have a formal relationship with any external organizations, agencies, institutions, or committees?
	Volume and type of proposals reviewed	How many research proposals were reviewed by the IRB in the latest year for which you have data?
	Membership	How many members sit on the IRB?
	Resources	Does the IRB have a dedicated meeting room to conduct protocol reviews?
	Training	Does the IRB provide research ethics training for its members?
	Review practices	Are 'minutes' taken during IRB meetings?
National Policies, Laws and Structures	National guidelines	Are there national guidelines addressing the ethical conduct of research with human subjects?
	National laws and regulations	Are there national laws or regulations in the country regarding the ethical conduct of research?
	National structures	Are there other institutions with IRBs in the country?
	National training, development and advocacy	Are there any established accreditation programs related to research ethics in the country?

We also developed qualitative data collection strategies, In-depth Interviews (IDI) and Focus Group Discussions (FGD) for the assessment. The IDI were meant to obtain information on the current research ethics system from individual stakeholders, as well as their personal experiences relating to research ethics training, resources, programs, and committees at the institution. We developed an interview guide for these semi-structured interviews, which lasted 45–60 minutes each. The in-depth interviews were done with 2 stake holders including one researcher and one graduate student. The FGDs were meant to understand the experiences and perspectives of specific groups of stakeholders who participate in the institutional research ethics systems including graduate students, research staff, researchers and members of the IRB. We developed a semi structured interview guide for the FGDs with appropriate flexibility for modifications for the different groups (Table 4). A total of four FGDs were conducted: one with 5 IRB members, one with 4 research staff, one with 5 graduate students, and one with 4 researchers.

**Table 4:** Key domains and target groups for In-Depth Interviews and Focus Group Discussions

**Domains**	**Target group for IDI/FGD**	**Illustrative question/s**
Research ethics influences	Research staff, researchers	How does research ethics affect your day to day research activities?
Research ethics training	Graduate students, research staff, researchers, IRB members	Have you ever taken any courses in bioethics or participated in ethics training?
Research ethics resources	Graduate students, research staff, researchers	If you had a bioethics or research ethics question, where would you go to get an answer?
Perception of strengths and challenges	Research staff, researchers, IRB members	What do you see as institution's greatest research ethics strengths?
Research ethics and your institutions approach to ethics review	IRB members	What do you feel is the role of the IRB at the institution?
Research experience	Graduate students	Have you conducted any research at this institution as a graduate student?
Relationship with IRB	Research staff, researchers, IRB members	How would you describe the IRB's relationship with researchers / research staff?
IRB Composition	IRB members	Where, if anywhere, is content-area expertise lacking in the committee?
Quality of IRB Review	IRB members	Do you feel the review process adds something to the research being conducted?

*IRB* Institutional Review Board (or Research Ethics Committees).

We also integrated a self-assessment in our approach. The octagon description along with an explanation of scoring was sent to the institution for their own ranking of performance on research ethics. This allowed for a comparative analysis of internal with external assessments and an opportunity for discussion around differences. In addition, the institutional leadership was asked to provide any written documents pertaining to policies on research ethics, application forms or checklists used by the IRB, research strategy, policies on ethical conduct, and guidelines for researchers on the kinds of research that needs to be submitted to the IRB for approval. All the documents provided by the university were then carefully examined for their content and consistency with each other and with the institution’s overall policy on research ethics and with responses made by leadership or individual respondents.

Our approach using multiple methods for collecting data described above is summarized in Table 5 and helped inform the institutional assessment for research ethics along the Octagon model that we present here. This approach is being tested by our FABTP in other countries and institutions as well.

**Table 5:** Summary of methods used for Institutional assessment of research ethics

	**Component**	**Description**	**Respondents**
1	Review of published documents	1.University Research Strategy	
		2.Policy on Ethics and Ethical conduct in research	
		3.Guidelines on the ethical conduct of research involving humans as participants at the University of Botswana	
		4.Application for IRB approval for research involving human participants	
		5.Checklist for ethical review by the IRB members	
2	Institutional Survey	Focuses on current research ethics capacity, organizational and operational structures, practices, training, policies, funding, and human resources.	Institutional leaders
3	In-depth interviews	Interviews conducted with the aim of exploring key facts and activities by institution in the areas of health research, research ethics and bioethics.	•Departmental heads
			•Research ethics committee chairs
			•Senior academics
			•Researcher representatives
4	Focus group discussions	To assess experiences with ethics training, ethics review procedures, and institutional relationships.	•IRB members
			•Researchers
			•Research staff
			•Graduate student representatives
5	Survey of IRB Representatives	An individual survey focusing on perception of IRB operations and needs	Members of the IRB

## Results

### The University of Botswana case study

In 2010, our FABTP partnered with the University of Botswana (UB) in Gaborone, Botswana. UB is the premier educational and research institution of the country, with approximately 15,700 students and 800 academic staff [25]. Our evaluation of the health research ethics capacity at UB in 2010 was conducted in accordance with the approach described above. That is, two site visits were made, and the institutional survey was completed by the university leadership. We also conducted IDIs and FGDs with institutional leadership, staff, faculty members, researchers, graduate students, and IRB members. All data collection was conducted in English, audio recorded and transcribed. All transcripts were read separately by three members of our research team (AAH, WZ, and JA) and thematic analysis performed and coded along the domains of the Octagon model. The institutional survey was also read carefully by all five internal reviewers (AAH, WZ, JA, RS, and NK) and the questions mapped onto the eight domains of the Octagon. Based on all of the data including site visits, a score was given for each of the eight domains of the Octagon; these scores represented a consensus amongst our research team. These scores by our team were then also compared with the scores provided in the self-assessment by the institutional leadership of UB including the director of the Office for Research and Development, the Assistant Director for Research Ethics, and a professor in the school of medicine.

A total of 18 participants were involved in the FGDs of whom 5 were members of the UB IRB, 4 were faculty researchers, 4 were members of research staff and 5 were graduate students. The mean age of the participants was 40 years. Twelve participants were female, 7 held a bachelor's degree, 2 had a master's degree and 9 had doctorates. Below we present results pertaining to each of the eight domains of the Octagon. These results reflect University of Botswana's research ethics capacity at the time of evaluation in 2010 only. This case study was approved by the institutional review boards of the Johns Hopkins Bloomberg School of Public Health, USA and the University of Botswana as well as the research ethics committee at the Botswana Ministry of Health.

### Basic values and identity

The University of Botswana has a strategic plan (updated in 2008) which addresses key policy areas including research and development, intellectual property, and ethics. In addition the university has written institution-wide policies that describe the kinds of research activities that need approval from the IRB, and what information researchers should include when submitting research protocols to the IRB. Notable strengths of UB in this regard are the formation of the Office of Research and Development (ORD), the wide range of protocols that need approval from the IRB, and the fact that policies related to research ethics are easily accessible to researchers through the university intranet. A primary goal of the university is to facilitate the development of new scientific knowledge by becoming a "research intensive" institution by the year 2021. In order to achieve this, the university has expanded its PhD program and facilitated coordination between the ORD and the graduate school.

However, while the ORD has clearly identified internal structure of authority and role regarding research ethics, the university needs to better identify how ORD relates to other departments and position the office clearly in an organizational chart. Possibly due to this lack of clarity, currently the role of ORD was not well understood by some researchers and students.

### Structure and management

UB has a clearly defined internal structure of responsibility and authority for research ethics under the ORD, which is overseen by a director who answers to the Deputy Vice Chancellor for Academic Affairs. All research ethics activities are directly supervised by an Assistant Director for Research Ethics. In highlighting the role of ORD in coordinating research ethics activities across the university, one informant explained:

“*[the] office is responsible for helping them [researchers] get those research permits [issued by Government] and the person responsible assists them to ensure that all the ethical issues have been clarified …. Usually when someone requests, or has an issue, they call and then we clarify those things and also have one on one session with people [researchers] who have issues and someone also goes to board and faculty meetings to have meetings with them”* (IDI-research staff).

In addition, several other committees relevant to the smooth, safe, and efficient conduct of research exist under the jurisdiction of ORD, including the UB Animal Care and Use committee, Hazardous Materials Sub-Committee, Research Risk Committee, and the Institutional Review Board. A strength of the IRB structure at UB is the existence of an explicit policy for gender representation, and IRB members are drawn from a wide range of backgrounds including religious and community representation. At the time of this institutional interview, there were 8 males (67%) and 4 females (33%) serving on the IRB. However, UB did not have an organizational chart showing how the ORD relates to other departments within the university.

### Implementation of Activities / Production

The teaching program at UB covers many of the substantive areas in research ethics; these are covered through specific sessions in existing research methods courses, requirements for student research, ad hoc training workshops by the ORD, and mentoring by UB faculty. However, ethics training is not an institutional requirement; rather, it is taught based on the perceived need and priorities of individual instructors. The need for more widespread and formal ethics training came up during the FGDs with graduate students as one student remarked:

“*There is not anything like that [ethics training], there is not any course in our program. The lecturer talked about it, gave scenarios I think trying to prepare us but it was not a real course ….. I think it would be good to put ethics in research as a course”* (FGD graduate student).

The FGD with the research staff also indicated that they felt inadequately trained in research ethics. For instance none of the staff interviewed were fully conversant with the concept of "informed consent". One participant pointed out that:

*“There was not any form of training as to what one should do when one meets the [research] communities. I think the reason why they gave me the job to run the program is the fact that I have done research before when I did my Masters Degree! That's how it was”.* (FGD research staff)

The IRB’s work at UB is both extensive and collaborative. In the year 2009 the IRB reviewed 50 proposals of which 33 were local, while 12 were in collaboration with other institutions. UB is registered with the United States Office for Human Research Protection and has a Federal Wide Assurance from USA. A challenge regarding the implementation of research ethics activities relates to workload and recognition for the work done by IRB members. Informants reported a perception that the load affected the quality of the review, and the turnaround period for protocols submitted. One IRB member said:

“*Sometimes we are really not in a position to offer justice [to reviews]…….we have no…..special discount somewhere that we can be given some time out from our heavy academic loads so that we can develop ourselves as [IRB] members and we can contribute in a productive sense…we have to face this…it is all up to us. So we need some organizational support….we need some real support”. (*FGD- IRB members*)*

Respondents also pointed out that the IRB currently has no expedited review option and even simple research proposals take a “long time” before approval. The respondents also stressed the need for the IRB to properly monitor ongoing research after they have been approved.

### Relevance of activities

UB’s Office of Research has stated its aims to increase capacity for ethical research among students and staff; and to ensure that human subjects research done at the university follows international guidelines for ethical research. Although the IRB at UB has been formally in existence for over five years, effectively it was reported to be functional only for the last two years. Owing to the large number of demands on committee members' time, the IRB has found it difficult to meet regularly. Some members felt that the IRB was "unconscious" in the early years since it managed to meet only twice a year. UB is working towards its goals regarding ethical research, and the constitution of the IRB and teaching of ethics to students have been ongoing for a few years. Some of the strengths of the UB IRB activities include the diversity of the committee in terms of representing different sectors and disciplines; its collaboration with outside groups and IRBs; its collaboration with other research and policy committees at UB; and good community involvement with representation of community members on the committee.

One particular challenge alluded to during several interviews was the low turnout for optional or voluntary ethics training activities. As a UB stakeholder commented:

“*Maybe we need to change some particular aspects on ethics training because the people that need it [the training] do not come. Those faculties [staff] that we’ve had troubles with on matters of human subjects’ protection are the very ones who are not coming. "* (IDI-Stakeholder)

This has led UB to consider instituting policies that would require researchers and graduate students to undergo some sort of research ethics training.

### Right skills for activities

Interviews with UB staff suggest that hiring for the ORD is a rigorous process and effort is made to hire people with appropriate skills and experience. However, for existing personnel serving in the ORD there are no requirements for professional training. UB faculty with significant interest in bioethics research and training do have relevant qualifications (one PhD in Law, and two faculty members with Masters degrees in Medicine). Two faculty members have publications on ethics-related subjects; however, only one faculty member had primary training in public health despite UBs interest in population research.

UB has written policies promoting training of IRB members but there are no minimum training requirements. The IRB provides short workshops and seminars on research ethics to members several times a year, and in 2009 four members of the IRB received such training. There is no requirement for refresher training for IRB members. However, participants stated that the short workshops offered by the IRB are insufficient:

“*It [the training workshop] is very fast and without any practical problems where you’re really given cases. Where you put your mind, your complete self, to discuss those cases to see how and what are the challenges?”* (IRB member)

“*I attended that [training workshop] three or four years back, but it was again a snapshot. It was not really what is going to give you a clear vision and a clear picture. It was just very fast without proper training*” (IRB member)

Another challenge faced by UB is a high proportion of guest workers and expatriates among the staff. Consequently there is a high turnover of the staff, often leading to a lack of consistency in research ethics activities. Another issue participants pointed out was the absence of a clinician serving on the IRB partly due to the fact that the school of medicine at UB was new and had a lot of staff turnover.

*"So what we do not have on this committee at the moment is a clinician, as such another representative who is on this committee but is absent at the moment who is not medically trained, but a basic scientist has been involved with a lot of human study"* (IRB member)

### Systems for financing and administration

Every year, UB allocates funds for supporting all research through its annual budget; this is a significant strength of UB and currently not reported from other African institutions. Such funds cover items such as salaries for ORD staff, internal research grants, and research support activities such as research training, research awards and others. This annual allocation is about 1.5% of UBs annual budget. UB is also able to raise funds from external sources and has research grants from for example the Wellcome Trust, the United States National Institutes of Health, and the European Union. The IRB at UB does not have an independent budget or an external source of funding. It is supported through the funds allocated for research support activities. IRB members are also not compensated for their services, however, and outreach programs or community mobilization programs by the IRB are sometimes stifled from lack of funding.

### Relations with target groups

UB identified the following target groups as important to the research ethics activities: human subjects (local community), students, faculty, health researchers, the Ministry of Health, the Government of Botswana and external collaborators. The UB has established an educational scholarship program in its effort to try and give back to the local community, as stated by this participant:

*“Issues of funding of course sometimes limit us, but we have done that much in terms of bringing them [community members] on board to have access to University, higher education. The research work that we are doing is to identify and see how we can reform policy and access of the course for populations or the communities we work with”* (FGD research staff).

Moreover, UB staff has operationalized this concern and addressed it and also identified staff to perform outreach programs:

*“I am [mentions his title], and well in terms of interaction with the people, from time to time I do what you refer to as outreach. In this I have to travel to the remote areas and meet the [names a minority group] and talk about the program and also the education of the people that we meet for our Scholarship Program and I will say well, in terms of how often I meet with the people, it also depends on the, on our budget".* (FGD research staff)

The focus group discussion with research staff indicated that at least some members of an indigenous community where UB has an ongoing study have complained that "you come here often and nothing comes to us":

*“There wasn’t any capacity development component. Later that came as a result of this research which showed that there are huge gaps in terms of access to resources, social services, even in education and so the University funded to put in the next component so that research was not just done on these communities." (*FGD research staff)

In addition the research staff highlighted the potential concern some of them had around undue influence when studies offer incentives for research participants. Respondents also reported that UB has good relations with the Ministry of Health, the government, and external collaborators.

### Working environment

This category reflects particularly positively on the UB’s pioneering role in developing health research ethics capacity in Botswana since there are no national guidelines for professional organizations or for human subjects research. The only research law in Botswana (as of 2010) dated from 1960s covering anthropological research. And even though other institutions in Botswana have IRBs, they do not offer ethics training programs.

During focus group discussions, several participants highlighted the regulatory overlap between the UB and Botswana's national ministries, particularly the Ministry of Health. As of 2010, research protocols must be approved both by the IRB of UB and by a ministry committee which issues a permit. Consequently researchers have to fill out multiple forms to submit to the IRB and ministry. This overlap between the function of the UB IRB and the ministry was reported to often cause inordinate delays before approval for research can be obtained. Concern was also voiced that the permit from the ministry, if obtained in advance, might be seen as influencing the deliberations of the UB IRB.

### Comparative assessment

**Figure 1:**
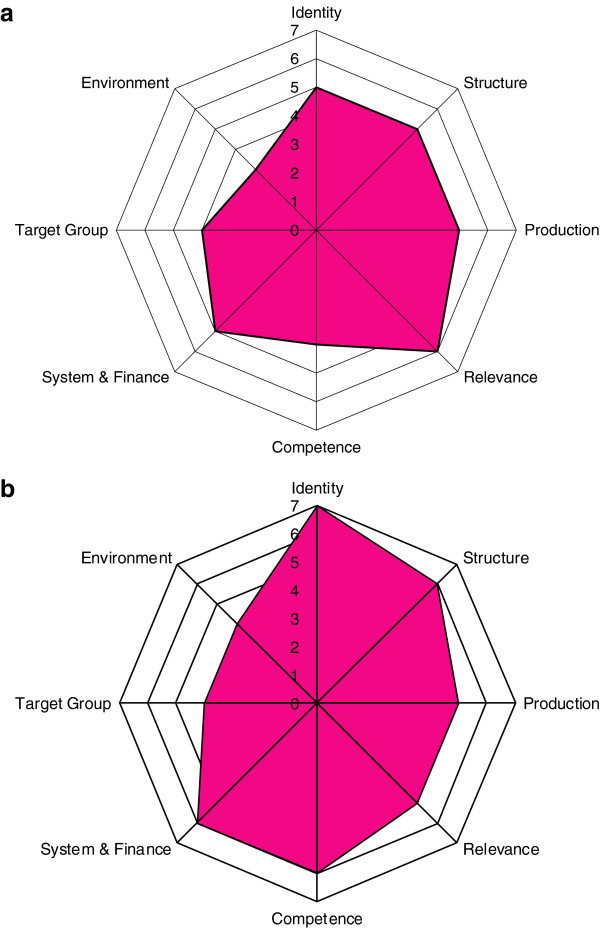
**Scored results of research ethics capacity assessment of University of Botswana. (a) **score by FABTP team, **(b) **Self-Assessment by University of Botswana.

Figure 1a & b shows the scored Octagons by both our team (external) and the internal UB groups – and demonstrates several interesting issues. The overall shape suggests that strengths at UB are in the areas of structure, relevance, production and identity; while UB still needs more work in the areas of systems of finance, target groups, and environment. The Octagons also show the similarities and discrepancies between the ‘external’ and ‘internal’ evaluations – which provides the opportunity to have specific discussions with UB to explore the reasons for these different assessments and why UB might be over estimating specific domains (or why we might be under estimating). For example, in this case study the score for ‘identity’ allows for an exploration of what constitutes a strong identity for research ethics at UB and how it can be strengthened and better documented. Such discussions are ongoing.

## Discussion and conclusions

In this paper we have described an approach to institutional assessment of research ethics capacity and share the initial results from a pilot study of the University of Botswana. The basic aims of such an institutional assessment are to establish an evidence-informed baseline for research ethics capacity for the future; to identify areas that need improvement or capacity building through partnerships; and to explore how different stakeholders view the research ethics system associated with the institution. To achieve this we have developed an operational approach to such assessments with the modification of an existing deliberative tool and hope that it can better inform such assessments. Initial testing of the approach is promising in a single case study and additional case studies are underway.

A foundational goal of UB is to become a “research intensive” university by 2021. This entails two commitments by the university; first that it will undertake pioneering research relevant to local needs, and second that it will ensure that this research is conducted while conforming to the highest ethical standards. This commitment to research ethics is unique in a number of ways. UB has developed institutional structures including a dedicated office and ethics committees for overseeing research ethics and has provided funding for the function of ORD and for the hiring of new staff into key positions, including those related to research ethics. The leadership of UB has shown strong support and encouragement for the development of research ethics capacity at the university. UB has a written strategic plan for developing research ethics capacity and has also developed institution-wide policies regarding research activities that need IRB approval.

This remarkable achievement for an African university has taken place against the backdrop of a challenging national context. Currently there are no national guidelines in Botswana specifically pertaining to research ethics and no laws that regulate the operation of interest groups that may influence the conduct of research. The regulation of human subjects research is, according to both institutional officials and other key informants, inadequately covered by the older law for anthropological research, though at the time of writing efforts are underway to draft a new law. There are no national training programs or accreditation processes for the constitution of IRBs. While human subjects oversight is provided by the Ministry of Health, there are no enforcement mechanisms to ensure adherence to those guidelines for ethical research. The experience of UB in strengthening its research ethics capacity in this context might not be representative of many other countries in sub-Saharan Africa; partly because of the university's ability to fund new structures relevant to research oversight and to attract highly trained (mostly African) expatriate faculty. Botswana's political stability and higher level of economic development may well also play an important role in an enabling national situation.

In addition to a challenging national context, there are several other significant areas for improvement. A robust and comprehensive training program in research ethics is crucial for capacity building in Botswana. A more systematic integration of research ethics training in student curricula and mandatory training for researchers at UB will be important steps in enhancing institutional capacity for research ethics. Similarly it is important to have explicit and enforced requirements for IRB members to have some training in research ethics; and this can be accomplished by short courses and refreshers. The use of personnel at UB and in the country, trained by FABTP and other Fogarty programs is an asset for this purpose. There also appears to be a need for wider dissemination among students and researchers of the IRB review processes and how to adequately meet the review requirements. Regular and structured communication between the IRB, researchers and students will foster trust and enhance the legitimacy of the IRB process. Another area that needs coordination is the process of national ethics review; while some of it lies beyond the control of UB, a streamlining of the review application process and current efforts to avoid duplicate submissions to UB and ministry of health will facilitate research and increase cooperation from researchers.

While remarkable structures for research ethics have been successfully put in place at the institutional level, the leadership of UB may want to build on this to increase the awareness of the UB research community in research ethics processes. Regularly conducted structured training, communication about the review processes, public demonstration of leadership support for research ethics activities, budgetary allocation to ensure sustainability, and greater harmonization of research ethics processes with the broader institutional activities are some ways in which "institutionalization" of research ethics system can be deepened [26,27]. Deeper institutionalization of the research ethics system is, in turn, expected to increase morale and job satisfaction of researchers and members of the IRB [28]. At a broader level, it is important for the research ethics system that the changes undertaken at UB are sustained through transitions in leadership; continuity in support and sustainability of university-supported initiatives often remains a challenge in many LMIC settings.

There is a general lack of frameworks for evaluating research ethics capacity in LMICs. We presented a case study based on the Octagon model, an approach that stresses evaluation from both internal and external perspectives. This multi faceted approach has several advantages including an extended interaction between the organization's leadership and the evaluation team, a focus on the institution and the context in which it operates, an opportunity for stakeholders to express their views on the strengths and weaknesses of the organization, and methodological triangulation which allows for a more complete picture to emerge than would be possible with the use of any one approach alone [29]. However, as always, it is possible that official self-assessment might be influenced by overly-optimistic perceptions or a willingness to sugarcoat reality. This is one of the reasons for our reliance on multiple methods to develop a more nuanced understanding of issues involved and to control for potential social desirability bias*.*

It is also important to highlight that, while informed by data, such an approach is fundamentally about tracking progress of an institution and not comparing across institutions (which is therefore a limitation). The derivation of a score on the Octagon is based on the team, time, interactions, information obtained, and a host of other factors – and should not be equated with the objectivity of a health statistic. Its value lies in its ability to stimulate institutions to think about how they operate and how they can improve themselves in the arena of research ethics. Such an approach is particularly suitable in situations where institutional data is traditionally scarce and the evaluation has to be done over a relatively short period of time. Another potential utility of this approach may be to inform training of research ethics committees and to focus their efforts on institutional needs. It is our hope that such studies in LMIC will lead to greater dialogue and development of a more holistic framework suitable for evaluation of research ethics capacity in resource poor settings.

Hyder et al. have suggested that a country's stage of development is related to its research ethics capacity by facilitating the emergence of national strategies, institutional commitments, and reviews of research ethics all of which influence the ethical conduct of researchers [10]. Future work is needed to better understand the challenges in developing research ethics capacity from a systems perspective in LMIC and what role institutions in high income countries can play in overcoming these challenges through collaboration and resource exchange. The current global focus on research ethics in many parts of the world provides an opportunity for a robust dialogue on ways to strengthen research ethics systems and to evaluate them in a consistent and transparent manner.

## Competing interests

'The authors declare that they have no competing interests.

## Authors’ contributions

AAH planned and conceptualized the study, was involved in data collection and analysis and drafted the manuscript. WZ was involved in data cleaning and analysis and drafted the manuscript. JA was involved in data collection, analysis and drafting of the manuscript. RS and PN were involved in data collection and drafting of manuscript. NK helped in conceptualization, data analysis and drafting of manuscript. All authors substantially contributed to drafting and revision of the manuscript and gave approval of this version.

## Pre-publication history

The pre-publication history for this paper can be accessed here:

http://www.biomedcentral.com/1472-6939/14/31/prepub
